# Richmond Academy of Medicine and Surgery

**Published:** 1896-04

**Authors:** Mark W. Peyser

**Affiliations:** Secretary and Reporter


### Richmond Academy of Medicine and Surgery, January 14, 1896.

The following officers were installed for the ensuing year: Dr. Landon B. Edwards, treasurer; Dr. Jno. N. Upshur, first vice president; Dr. J. P. Massie, second vice president; Dr. J. W. Henson, third vice president; Dr. Mark W. Peyser, secretary; Dr. Wm. R. Jones, assistant secretary; Dr. R. A. Nichols, librarian.

Dr. G. W. Le Cato, an invited guest, read a paper on "The Continued Fevers of the Eastern Shore of Virginia."

### DISCUSSION.

Dr. John N. Upshur said the paper was characterized by good, hard, common sense, and there was very little with which he could take issue. He is in accord concerning the germ theory; but believes typhoid is contagious as well as infectious, and referred to one case infecting every one who into contact with her, until twenty were affected. He believes he contracted the disease immediately from his mother. Dr. Upshur said he was interested in the statement that bilious fever has been replaced by endemic typhoid on the eastern shore, and thinks it due to the removal of forests, and consequent free ventilation from salt water; but he does not see why typhoid should exist there, taking into consideration the care taken with farm houses and surroundings. He is in accord with Dr. Le Cato concerning treatment, especially with regard to strychnine. The Woodward treatment, the philosophy of which the doctor said he could not understand, was referred to.

Dr. J. S. Wellford said he was satisfied that there is such an entity as typho-malarial fever.

Dr. Arthur Jordan stated that in typhoid there were certain indications in the blood in the first week—absence of leucocytosis. In the second week, there is a leucocytosis, but it differs from that of other acute inflammations in that it is a lymphocytosis. In malaria, there is always the plasmodium. Therefore, it seems that a microscopic examination of the blood will demonstrate whether or not there is typho-malaria.

Dr. J. P. Massie inquired if Dr. Jordan meant the disease was a hybrid.

Dr. Jordan responded that the disease known as typho-malaria has not brought out any distinct appearance. Investigations have failed to show that the plasmodium and Eberth's germ are present at the same time, and exerting at the same time their specific influences.

Dr. W. S. Gordon thinks Dr. Le Cato has arrived at the practical point. His studies have shown that where there is a dearth of civilization, there is malaria; but as soon as the country becomes settled and drained, typhoid appears. Therefore, we must look to civilization for the cause. It is hard to understand how two specific germs, acting at the same time, can produce a modified disease. He grants that one can have two specific fevers at the same time; but why do we not have the specific manifestations of typhoid and malaria simultaneously. Chills may be seen in true typhoid. We are bound to the fact, that we must establish the main symptoms, e. g., recurring chill of malaria. Osler has established the fact, that the so-called typho-malaria is typhoid.

Dr. Upshur brought up the question of contagion. If the disease is of germ origin, it must be granted that it can be conveyed by air or water. He is rapidly coming to the belief that it is a water-borne disease, and we must look to fluids as the carriers. Dr. Gordon referred to an epidemic occurring twenty-one days after partaking of water from a well he had condemned. Concerning treatment, he is of the opinion that in spite of Pepper's nitrate of silver, and others, we have not yet arrived at any specific course.

Dr. Hugh M. Taylor stated that he had failed to observe in atypical typhoid fever, any manifestation to justify the suspicion that it was in any way related to malaria. Certainly, in his hands, such acute malarial remedies as quinine, not only did no good, but did harm. He thought everything tended to show that the term, typho-malarial fever, is a misnomer. He is wedded to the idea that the poison of typhoid is water—or milk-borne, the exceptions being so few that they are not worth considering.

As far as his observations go, there is very little well-manifested malaria in Richmond, and he does not think he sees one case of chills and fever a year; but we do have our share of typical, as well as atypical, typhoid.

As to the possible existence of two specific germs, existing and operating at the same time in patients, he does not think it impossible. In exceptional instances in typhoid, there is developed an osteo-myelitis, limited, as a rule, in extent, with a strong tendency to symmetrical development, and to suppuration. Whence comes this pyogenic micro-organism? Has the typhoid bacillus, under changed environment, acquired pyogenic properties, or has there been created, by the systematic depression of typhoid, a *locus minoris resistential*, and a suitable point for the lodgment and morbific action of pyogenic organisms, brought to the point of lessened resistance by the blood? It is known that one can have typhoid and pneumonia; can have the product of the tubercular bacillus, infected by pyogenic matter, and both will contain material potent for local and systemic injury to the tissues. Infective arthritis exceptionally occurs in connection with typhoid fever, as well as other specific fevers. This inflammation may, or may not, go on to suppuration. If it does, is the suppuration due to pyogenic properties assumed by the specific germ of the disease, or to pyogenic microbes imported from without, and transported to the joint by the blood; or is it a result of the combined action of the two organisms? This is an unsettled problem, but in view of the fact that the typhoid bacillus, and the common bacillus of the colon, have so many points in common, that the doctor does not see why the typhoid bacillus may not, under changed environment, assume pyogenic properties, as well as the bacillus coli communis.

Dr. Jacob Michaux asked, may not the disease be entirely distinct from either typhoid or malaria? He is very much disposed to believe it is a different affection, looking at it from either point of view. In treatment, he thinks water is of great value in depuration; and he is surprised at the injunctions limiting it. It is the medium of exchange in the body, and plays a great part in elimination. Taken internally, or used locally, it acts upon the skin, reducing temperature. Strychnine is a decided advance in treatment.

Dr. Upshur remarked that one may have typhoid fever in a malarial district, the latter impressing itself upon the case. Post-mortems show how malaria affects the ulcerations of the bowels, the edges having characteristics almost distinctive.

Dr. Le Cato, in concluding, said he was glad his paper had brought out so many points, and had directed the discussion into such various channels. He remarked that each case of typhoid doesn't present all the symptoms, but taking them as a whole, we have a complete picture. Variations of exceptions do not vitiate under the rule, and it is unfair to assume that because of a mild attack, the same poison does not lie at the cause. In his country, the prodromata are those of typhoid—slowly increasing temperature, tendency to hemorrhage, tympanitis, especially the latter. In these cases, we have an accurate description of typhoid. He has never yet been able to learn that the germs of typhoid and malaria have been demonstrated to exist at the same time. He thought he had satisfied himself beyond cavil, that typhoid is water-borne, but late observations have shaken his belief. He cited a case in which the patient refused, for two months, to use unboiled water, and had quarantined her residence, and yet she contracted typhoid. Other instances occurred in a house situated on the river's edge, on a sloping bank, water being supplied by a driver pump thirty feet deep. Tenement houses were situated lower down. These escaped, while the former was affected.

Mark W. Peyser, M. D.,
Secretary and Reporter.

